# Activation of homologous recombination DNA repair in human skin fibroblasts continuously exposed to X-ray radiation

**DOI:** 10.18632/oncotarget.4946

**Published:** 2015-08-13

**Authors:** Andreyan N. Osipov, Anna Grekhova, Margarita Pustovalova, Ivan V. Ozerov, Petr Eremin, Natalia Vorobyeva, Natalia Lazareva, Andrey Pulin, Alex Zhavoronkov, Sergey Roumiantsev, Dmitry Klokov, Ilya Eremin

**Affiliations:** ^1^ State Research Center - Burnasyan Federal Medical Biophysical Center of Federal Medical Biological Agency (SRC-FMBC), Moscow 123098, Russia; ^2^ Semenov Institute of Chemical Physics, Russian Academy of Sciences, Moscow 119991, Russia; ^3^ Dmitry Rogachev Federal Research Center of Pediatric Hematology, Oncology and Immunology, Moscow 117997, Russia; ^4^ Moscow Institute of Physics and Technology, Dolgoprudny, Moscow Region 141700, Russia; ^5^ Emanuel Institute for Biochemical Physics, Russian Academy of Sciences, Moscow 119991, Russia; ^6^ Insilico Medicine, Inc, ETC, Johns Hopkins University, Baltimore, MD 21218, USA; ^7^ The Biogerontology Research Foundation, BGRF, London W1J 5NE, UK; ^8^ N.I. Pirogov Russian National Research Medical University, Moscow 117997, Russia; ^9^ Canadian Nuclear Laboratories, Chalk River, ON K0J1P0, Canada

**Keywords:** DNA DSB repair, homologous recombination, human fibroblasts, X-rays, continuous irradiation

## Abstract

Molecular and cellular responses to protracted ionizing radiation exposures are poorly understood. Using immunofluorescence microscopy, we studied the kinetics of DNA repair foci formation in normal human fibroblasts exposed to X-rays at a dose rate of 4.5 mGy/min for up to 6 h. We showed that both the number of γH2AX foci and their integral fluorescence intensity grew linearly with time of irradiation up to 2 h. A plateau was observed between 2 and 6 h of exposure, indicating a state of balance between formation and repair of DNA double-strand breaks. In contrast, the number and intensity of foci formed by homologous recombination protein RAD51 demonstrated a continuous increase during 6 h of irradiation. We further showed that the enhancement of the homologous recombination repair was not due to redistribution of cell cycle phases. Our results indicate that continuous irradiation of normal human cells triggers DNA repair responses that are different from those elicited after acute irradiation. The observed activation of the error-free homologous recombination DNA double-strand break repair pathway suggests compensatory adaptive mechanisms that may help alleviate long-term biological consequences and could potentially be utilized both in radiation protection and medical practices.

## INTRODUCTION

Ionizing radiation exposure leads to a variety of DNA lesions, but the fate of the cell is largely determined by DNA double-strand breaks. These potentially lethal lesions are thought to be triggers of cellular responses to irradiation [1–3]. While the absolute number of DNA double-strand breaks per radiation dose unit is relatively low, estimated to be 20–40 breaks per cell per Gy [4, 5], the repair of DNA double-strand breaks is a slow and complex process involving tens of various proteins, spanning large areas of chromatin and affecting its conformation [6]. Misrepair or failure to repair a DNA double-strand break may lead to cytogenetic abnormalities, cell death, inactivation of tumor suppressor genes or activation of oncogenes [7–9].

Formation and repair of DNA double-strand breaks upon acute radiation exposures is relatively well characterized (e.g. see a review by Thompson [6]). In general, DNA double-strand breaks can be repaired by one of the two major mechanisms: non-homologous end-joining (NHEJ) or homologous recombination (HR). NHEJ repair, involved in an estimated ∼80% of DNA double-strand breaks [6], is cell cycle independent and fast, taking approximately 30 minutes to complete [10]. However, NHEJ is error-prone and can lead to various genetic abnormalities [11]. In contrast, HR repair is error-free and slow (> 7 h [10]) and requires a sister chromatid as a template for DNA synthesis in the vicinity of a break on the damaged chromatid. Therefore, this pathway is active mainly in cells in S and G2 cell cycle phases [12]. HR is also specifically involved in the repair of collapsed replication forks [13]. It is, therefore, important to know relative contributions of the two DNA double-strand break repair pathways in order to better predict or understand delayed consequences of exposure to radiation.

Although repair of radiation-induced DNA double-strand breaks is well studied for acute irradiation, responses to DNA double-strand breaks produced by continuous or chronic exposures to ionizing radiation are not well characterized [14–17]. Yet, most of the time, human cells are exposed to ionizing radiation chronically at low dose-rates, and possible health consequences of such exposures are of great concern [18–20]. The challenges for such continuous irradiation studies include both technical ones related to irradiation facilities and difficulties in interpreting results. For example, one would need to account for i) two opposite but concurrent processes of accumulation and elimination of DNA damage during exposure, ii) cell cycle redistribution, and iii) cell proliferation during exposure, i.e. a dose is split between mother and daughter cells, etc.

Advances in understanding molecular mechanisms of DNA double-strand break repair provide powerful experimental tools. In particular, these include identification of individual proteins or protein post-translational modifications that form complex dynamic structures in the vicinity of individual double-strand breaks. There may be thousands of copies of those molecules involved in processing of a single DNA double-strand break. Immunofluorescent labeling of such proteins makes possible microscopic visualization of the DNA repair structures as distinct spots or foci that typically correspond to individual DNA double-strand breaks [21–23]. This allows for very accurate and sensitive indirect quantification of DNA double-strand breaks and their repair, thus facilitating examination of molecular mechanisms of the repair process. By far the most common marker of DNA double-strand breaks is phosphorylated histone H2AX, called γH2AX [24]. H2AX is phosphorylated by ATM, ATR or DNA-PK kinases in response to DNA double-strand break formation and signifies the recognition of a break [25]. To examine the involvement of HR in the repair process, foci formed by the HR core component, RAD51 protein, are typically measured [26–28].

Given the importance of DNA double-strand breaks and their processing for health outcomes and the lack of clear understanding of how cells respond to chronic irradiation, we sought to measure formation of DNA double-strand breaks and the rate of HR in human diploid fibroblasts upon continuous irradiation in this study. We quantified total number of DNA double-strand breaks using γH2AX foci and in parallel, evaluated the extent of HR repair by measuring RAD51 foci.

## RESULTS

### Increase in number of γH2AX foci

**Figure 1:**
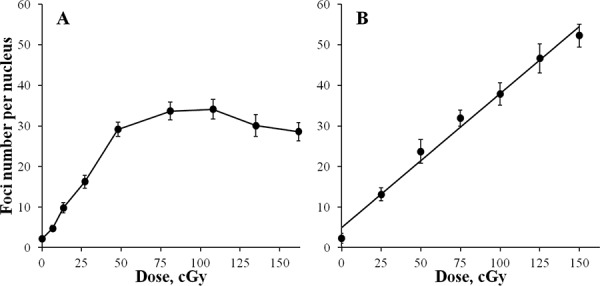
Formation of γH2AX foci in diploid normal human fibroblasts during continuous exposure to X-ray radiation at a dose-rate of 4.5 mGy/min A or 30 min after acute X-ray irradiation **B.** γH2AX foci were quantified using immunofluorescence microscopy. Two hundred cells per data point were analyzed per experiment. Means calculated from three independent experiments ± standard errors are shown.

Primary cultures of diploid human fibroblasts were exposed to continuous X-ray radiation at a dose-rate of 4.5 mGy/min under normal growth conditions for up to 6 h. At various times from the exposure start, cells were fixed and immuno-fluorescently labelled for γH2AX and RAD51. Kinetics of γH2AX foci formation is shown in Figure 1A. An increase in the foci number was detected at the earliest time-point of 15 min from the start of exposure corresponding to a 67.5 mGy cumulative dose. The number of γH2AX foci continued to increase linearly with exposure time for up to 2 h (540 mGy). The curve of γH2AX foci accumulation could be best fit with a linear function y = (2.24 ± 0.48)+(50.41 ± 1.72)x (*R* = 0.998, *p* = 0.0001, R^2^ = 0.996), where y is γH2AX foci/nucleus, x is dose in Gy. The number of γH2AX foci per Gy could be easily calculated from this relationship and was 50.41 ± 1.72.

Continuation of irradiation beyond 2 h did not result in a statistically significant increase in γH2AX foci (Figure 1A). Although, for 3 and 4 h time-points (0.81 and 1.08 Gy, respectively) higher values of γH2AX foci were recorded compared with 2 h, they were not significant and were followed by a slight decrease at 5 and 6 h (1.35 and 1.62 Gy, respectively). Therefore, we observed a plateau at about 30 foci per cell that persisted from 2 h untill at least 6 h of exposure.

To verify that the plateau was not a methodological artefact, we generated a dose-response curve using acute irradiation (Figure 1B). Cells were exposed to X-ray radiation at a dose rate of 400 mGy/min and fixed at 30 min post-irradiation when the maximum number of γH2AX foci is typically observed. A linear dose-response curve (y = (4.96 ± 1.44)+(33.00 ± 1.59)x (R = 0.994, *p* =0.000001, R^2^ = 0.989)) in the entire range of doses used up to 1.5 Gy was observed with the number of γH2AX foci increasing up to 50 foci/cell, which is well above the 30 foci/cell value observed for the continuous irradiation plateau. Thus, the lack of a dose-dependent increase in the number of γH2AX foci registered between 2 and 6 h of continuous irradiation was not due to method limitations.

### Increase in integral γH2AX fluorescence intensity

**Figure 2:**
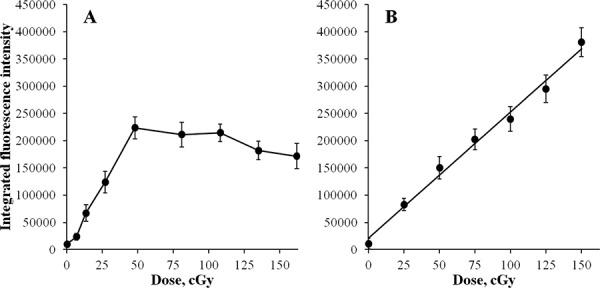
Changes in integral γH2AX fluorescence in diploid normal human fibroblasts during continuous exposure to X-ray radiation at a dose-rate of 4.5 mGy/min A or 30 min after acute X-ray irradiation **B.** γH2AX fluorescence was measured using immunofluorescence microscopy. Two hundred cells per data point were analyzed per experiment. Means calculated from three independent experiments ± standard errors are shown.

As the number of double-strand breaks grows with time of exposure, the probability of two individual breaks arising within close proximity and appearing as a single γH2AX focus increases. This may lead to underestimation of the real number of γH2AX foci and the appearance of the plateau on the curve that was observed between 2 and 6 h in Figure 1A. However, by measuring integral fluorescence intensity, as opposed to foci number, the contribution of this factor in the resulting kinetics of γH2AX foci accumulation may be examined. Results of such measurements are presented in Figure 2A. The initial phase of linear accumulation of integral γH2AX fluorescence with time (dose) up to 2 h was followed by slight decreases of fluorescence at 3, 4, 5 and 6 h compared with that observed at 2 h. As in the case of γH2AX foci number, the changes after 2 h were not statistically significant. We also plotted a dose-response curve for acute irradiation for integral γH2AX fluorescence and found a linear relationship that spanned up to 370,000 fluorescence units, which substantially exceeds the value of 230,000 units at which the plateau was observed for continuous irradiation (Figure 1B). Thus, both quantification of foci number and measurement of integral fluorescence used in this study have a wide linear range that is sufficient for reliable analyses of chronically irradiated cells.

### Increase in number of RAD51 foci

**Figure 3:**
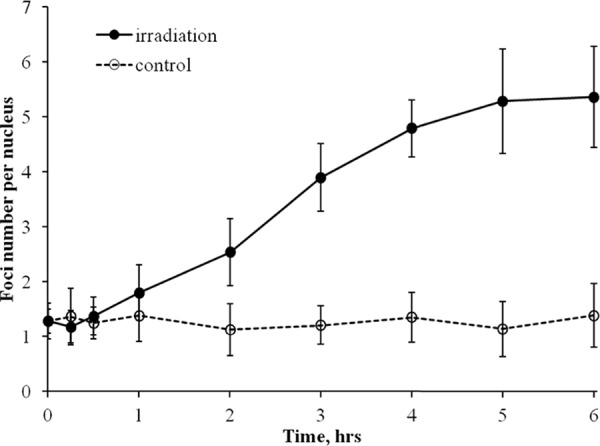
Formation of RAD51 foci in diploid normal human fibroblasts during continuous exposure to X-ray radiation at a dose-rate of 4.5 mGy/min RAD51 foci were quantified using immunofluorescence microscopy. Two hundred cells per data point were analyzed per experiment. Means calculated from three independent experiments ± standard errors are shown.

To evaluate a contribution of HR repair relative to total DNA double-strand break repair induced by chronic irradiation, we measured foci number and intensity for a key component of HR, RAD51. Normal human skin fibroblasts were exposed to 4.5 mGy/min X-ray radiation for up to 6 h and RAD51 foci were quantified as described in Methods. We observed increases in the number of RAD51 foci with time of exposure to X-ray radiation (Figure 3). Statistically significant changes were observed at 2 h of exposure and foci continued to accumulate up to 6 h. This was in contrast to γH2AX foci whose number did not increase beyond 2 h of exposure.

### Increase in integral RAD51 fluorescence intensity

**Figure 4:**
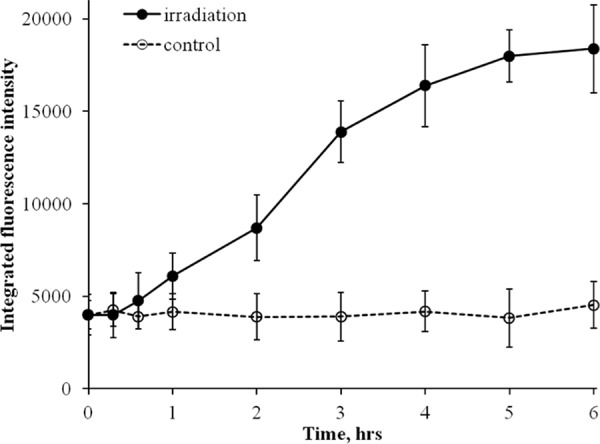
Changes in integral RAD51 fluorescence in diploid normal human fibroblasts during continuous exposure to X-ray radiation at a dose-rate of 4.5 mGy/min RAD51 fluorescence was measured using immunofluorescence microscopy. Two hundred cells per data point were analyzed per experiment. Means calculated from three independent experiments ± standard errors are shown.

Similar to γH2AX analyses, to better understand the kinetics of quantified RAD51 foci, we also measured integral fluorescence intensity of RAD51. The kinetics curve for integral RAD51 foci fluorescence intensity (Figure 4) mimicked the one obtained for RAD51 foci number (Figure 3), indicating that the lower rate of RAD51 foci accumulation between 2 and 6 h, as in the case for γH2AX foci, was not due to overlapped or merged foci.

**Figure 5:**
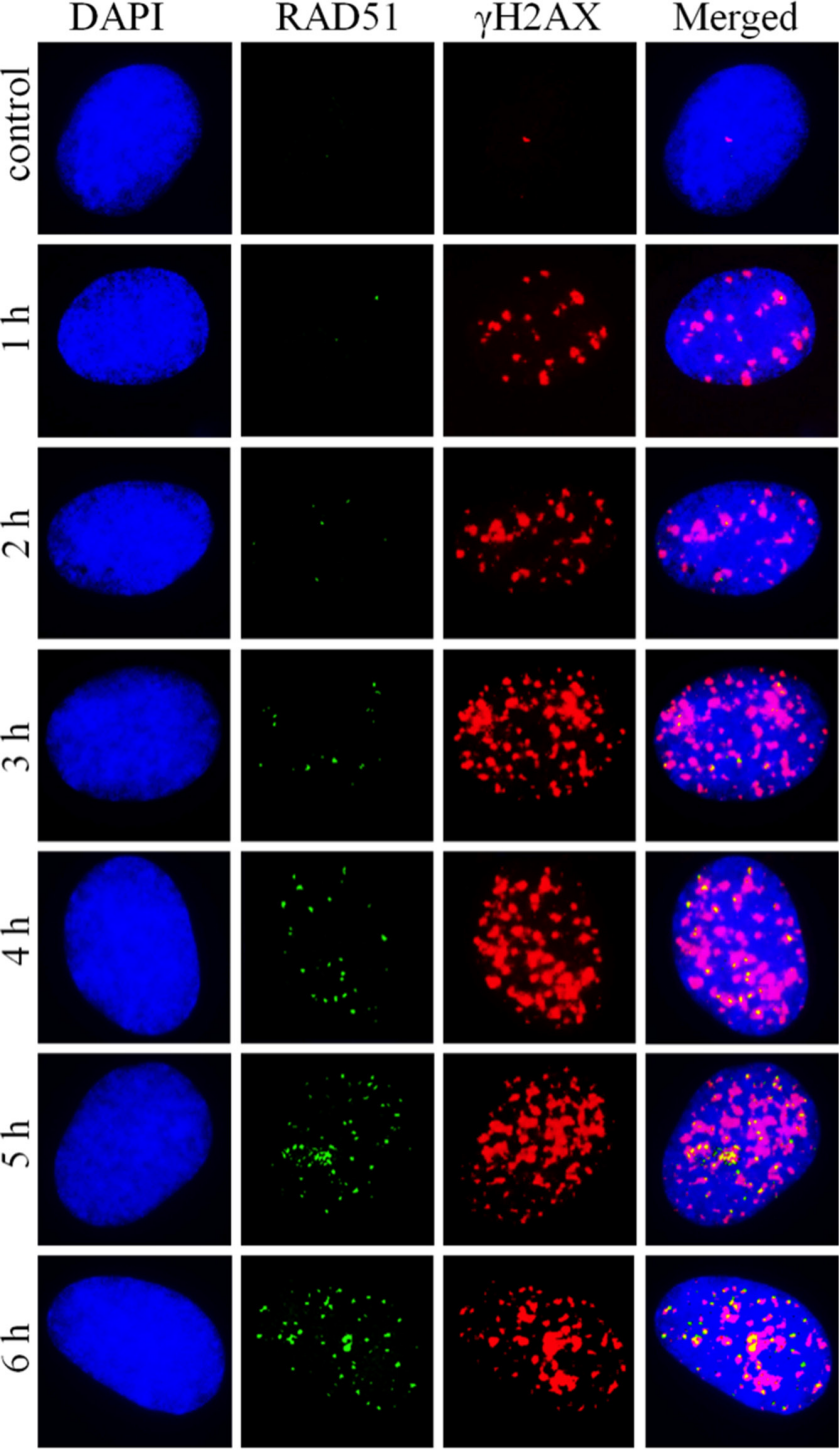
Representative microphotographs of RAD51 and γH2AX foci formed in diploid normal human fibroblasts upon exposure to X-ray radiation at a dose-rate of 4.5 mGy/min

Shown in Figure 5 are representative microphotographs of γH2AX and RAD51 foci obtained for various radiation exposure times. They illustrate the findings that γH2AX foci accumulate faster, but plateau at around 2 h of exposure, in contrast to RAD51 foci that accumulate initially slower compared with γH2AX foci but do not plateau up to the latest time-point of 6 h.

### Increase in RAD51 is not attributable to S/G2 cell cycle arrest

**Figure 6:**
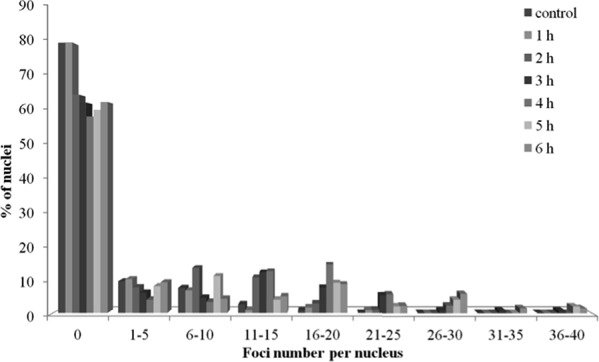
Distribution of diploid normal human fibroblasts with various numbers of RAD51 foci upon exposure to X-ray radiation at a dose-rate of 4.5 mGy/min for indicated periods of time

One important difference between RAD51 and γH2AX foci, within the context of this study, is that the former foci form predominantly in S/G2 cells, whereas the latter form in cells in any phase of the cell cycle. Thus, it was reasonable to assume that the increase of RAD51 past 2 h exposure time, with no further increases in γH2AX within the same time frame, may be indicative of the S/G2 cell cycle arrest, rather than enhanced HR repair pathway. To test this assumption we examined the distribution of cells with various RAD51 foci numbers (Figure 6). We found that by 2 h of radiation exposure the fraction of cells without RAD51 foci, typically representing G0/G1 cells, decreased to ∼65% from ∼80% in control un-irradiated cells. Further irradiation up to 6 h did not result in a further decrease in the fraction of cells without RAD51 foci, indicating that cell cycle distribution of cells did not change between 2 and 6 h of exposure.

## DISCUSSION

Radiotherapy is a key treatment for cancer. However, exposure of normal cells to ionizing radiation is associated with known health risks, such as cancer. While molecular and cellular response to acute radiation exposure is well-studied, fewer studies have examined responses to continuous or chronic exposures. In this study, we sought to determine the effects of continuous exposure to ionizing radiation on DNA double-strand breaks and repair. Using normal human skin fibroblasts, we measured the effects of a continuous 6-h exposure using X-ray radiation at a dose rate of 4.5 mGy/min on DNA double-strand breaks. We used two markers to measure the effects: γH2AX, which indicates a total number of DNA double-strand breaks, and RAD51, a marker of the slow, but error free HR repair process. We found that both the number and intensity of γH2AX and RAD51 foci increased over time; however, the kinetics of these increases differed markedly. While γH2AX foci increased linearly over the first 2 h of exposure, they plateaued thereafter. RAD51 foci, on the other hand, continued to increase throughout the 6-h irradiation experiment. In the following, we discuss these results and the hypothesis that these findings indicate an adaptive repair response to continuous radiation exposure that reduces the risk of long-term damage to the cell.

### Increase in γH2AX foci

Our results indicate that kinetics of DNA double-strand break formation upon continuous exposure to X-ray radiation monitored in normal human fibroblasts using γH2AX foci consists of two components: i) linear accumulation with time (dose) of exposure, and ii) plateau. The first linear component observed between 0 and 2 h of irradiation reflects accumulation of DNA double-strand breaks and somewhat slower repair compared with the breaks produced by acute irradiation. As a result, the number of DNA double-strand breaks or γH2AX foci within the first 2 h of irradiation per cell per Gy was unexpectedly higher than that observed in human fibroblasts exposed to acute irradiation (∼50 foci/cell in this study compared with ∼36 foci/cell in [25]). This observation, at first counter-intuitive, could be explained by a longer average life span of γH2AX foci upon chronic radiation exposure compared with acute irradiation, resulting in faster accumulation of foci. Indeed, depending on the complexity of the double-strand break, repair time may vary between minutes and hours [5]. Besides, the number of γH2AX foci per Gy obtained in our study is consistent with the number of ATM foci co-localized with γH2AX foci in a study by Suzuki et al. [29].

### Increase in γH2AX intensity

As the number of γH2AX foci accumulates with time/dose of exposure, newly formed foci can potentially overlap with the existing foci. In this case, discrimination of individual foci represents a significant technical challenge. Additionally, metabolic processing of damaged chromatin may lead to foci clustering within decondensed chromatin [30] or so called repair factories [31]. All these processes can result in underestimating foci number. The intensity of γH2AX foci, in these cases, would provide a more accurate evaluation of DNA double-strand break accumulation. As evident from Figure 2A showing the kinetics of γH2AX foci intensity, the lack of γH2AX foci accumulation beyond 2 h in our experiments was not caused by the process of foci overlapping and merging. Additionally, we show that our method of γH2AX foci quantification (Figure 1B) and measuring total γH2AX fluorescence (Figure 2B) has a wide linear range of detection that exceeds substantially the plateau values, validating our continuous irradiation results. Thus, the observed kinetics of the γH2AX end-points is an accurate representation of DNA double-strand break formation and repair upon continuous X-ray irradiation.

### Increases in RAD51 foci and intensity

When interpreting results of γH2AX measurements, specifically in terms of potential long-term biological outcomes, such as genetic instability and cancer risk, it is important to consider that the disappearance of a γH2AX focus technically means only a completion of ligation of two double-stranded DNA ends. This rejoining may be completed with substantial errors leading to gross cytogenetic abnormalities, such as dicentrics, rings, etc. The predominant pathway for DNA double-strand break repair, specifically at high dose-rate exposures, is NHEJ, which is prone to loss of genetic information. The loss could be very substantial in cases when overhangs on the ends of double-strand breaks are not homologous (microhomologies) and may reach millions of nucleotides and lead to deletions [32]. It is, therefore, advisable in DNA repair studies, if estimates of potential biological consequences are to be made, to not only measure the rate and completion of rejoining of DNA double-strand breaks, such as those provided by γH2AX assays, but also to evaluate contribution of the HR repair pathway. The ratio of NHEJ to HR components was shown to be a very important prognostic factor in irradiated cell populations that correlates with the radiosensitivity of cells [33].

In our experiments, both the number and intensity of RAD51 foci, indicative of HR activity, increased within the entire irradiation time period of 6 h (Figures 3 and 4). This is in contrast to γH2AX data that showed increases only within the first 2 h of irradiation. The increases in RAD51 foci number and their intensity per cell were not caused by an accumulation of cells in S/G2 phases of the cell cycle, as evident from Figure 6. Instead, they should be attributed to an increased number of DNA double-strand breaks that are repaired by the HR pathway relative to the total number of DNA double-strand breaks. It should be noted that the enhancement of HR might be a result of higher rates of secondary double-strand breaks within collapsed replication forks. Overall, our data indicate that at some point during continuous radiation exposure, activation of the HR repair of DNA double-strand breaks is triggered in normal cells by as yet unknown mechanisms.

### Adaptive response hypothesis

Firstly, the presence of a plateau in the kinetics curve of γH2AX foci/intensity, assuming they represent DNA double-strand breaks, can be considered as an activation of end rejoining or repair. Interestingly, similar results were obtained for low dose-rate irradiation in our recent study in immortalized Chinese hamster V79 fibroblasts [34], as well as for acute irradiations in earlier studies using indirect DNA damage end-points [35]. Inducible DNA repair was shown to be involved in such dose-responses [35]. Thus, the plateau observed in this study for γH2AX/DNA double-strand breaks may also be thought of as an adaptation process typically observed after low doses of acute irradiation (<200 mGy).

Secondly, the qualitative changes in the way DNA double-strand breaks are repaired past 2 h of exposure, seen as an activation of the HR pathway, can further be considered through a prism of adaptive changes. Indeed, with the HR pathway being less prone to errors compared to the NHEJ repair pathway that may result in point mutations and gross chromosomal aberrations [36, 37], the observed activation of HR may alleviate potential long term detrimental outcomes of irradiation, such as genetic instability and cancer. In line with this, it was shown that TERT-immortalized human fibroblasts respond to low dose-rate irradiation (0.3 mGy/min) with lower yields of chromosomal abnormalities and cell death per unit dose compared with high dose-rate (2000 mGy/min) irradiation [38]. Similar results have been reported for animal studies [39–41]. Interestingly, activated HR repair of DNA double-strand breaks enhances survival not only in normal, but also in tumor cells. Increased levels of RAD51 have been observed in tumor cells and were associated with better survival after radiotherapy treatments; thus, RAD51 can be considered as one of the targets to radiosensitize tumor cells [42–44].

In summary, our results provide an insight into the mechanisms of DNA double-strand break formation and repair upon continuous exposure to X-ray radiation in normal human cells. The demonstrated activation of DNA double-strand break repair and the found predominant role of the HR pathway in this repair may be utilized in various medical practices and help improve management of health risks associated with continuous exposure to ionizing radiation.

## MATERIALS AND METHODS

### Fibroblast cultures

All reagents were purchased from Sigma-Aldrich (USA) and cell culture plasticware was purchased from Corning-Costar (USA), unless otherwise stated. Experiments were carried out using primary human normal fibroblasts derived from skin biopsies from healthy volunteers (males, 50–52 years of age). After obtaining informed consent, 2 × 2 mm skin biopsies were excised from the area behind the ear under local anesthesia with 2% lidocaine. The biopsy material was placed in Dulbecco's Modified Eagles Medium (DMEM) supplemented with 5% Fetal Bovine Serum (FBS; Biological Industries, Israel), 1 g/L D-glucose, 100 U/mL penicillin and 100 U/mL streptomycin, and immediately transported to cell culture laboratory. Following the treatment with collagenase type II, cell suspensions were incubated for 14 h under 37°C and 5% CO_2_ in high glucose (4.5 g/L) DMEM supplemented with 20% FBS, 2 mmol/L L-glutamine (StemCell Technology, USA) and antibiotics. Cells were then detached using 0.05% trypsin in EDTA (StemCell Technology, USA) and seeded at 10^4^ cells/cm^2^ in DMEM supplemented as in the previous step with the exception of lower FBS concentration (10%). Fibroblasts were expanded by sub-culturing at 80–90% confluency, with medium changed every 3 days. Cells were then detached, frozen and stored in liquid nitrogen in complete DMEM supplemented with 10% DMSO. For experiments, cryovials with cells were quickly defrosted in 37°C water bath and complete DMEM was slowly added to cells to a total volume of 50 mL. A small aliquot of cells was used to determine cell viability using Countess cell counter (Invitrogen, USA) according to the manufacturer instructions. For all experiments, viability was ≥ 92%. Three days later, exponentially growing cells (∼70% confluency) were trypsinized and seeded onto coverslips (SPL Lifesciences, South Korea) placed inside 35 mm petri dishes. Next day cells attached to coverslips were exposed to X-ray radiation. Acutely irradiated cells were returned to a CO_2_ incubator for 30 min followed by a fixation.

### Irradiation

For protracted irradiation, cells were exposed to 50 kV X-rays at a dose rate of 4.5 mGy/min (0.4 mA, 1.5 mm A1 filter) using RUB RUST-M1 X-irradiator (Russia). Throughout the irradiation, cells were maintained at 37°C using a heated stage with a thermo-regulator. Acute irradiation was carried out on ice using the same device and the dose rate was 400 mGy/min.

### Foci detection and analysis

Cells were fixed on coverslips in 4% paraformaldehyde in phosphate buffered saline (PBS, pH 7.4) for 20 min at room temperature followed by two rinses in PBS and permeabilization in 0.3% Triton-X100 (in PBS, pH 7.4) supplemented with 2% bovine serum albumin (BSA) to block non-specific antibody binding. Then, cells were incubated with primary mouse monoclonal antibody against γH2AX (05–636-I clone JBW301, Merck-Millipore, USA) and rabbit polyclonal antibody against RAD51 (ABE257, Merck-Millipore, USA) diluted in PBS (1:400 and 1:200, respectively) with 1% BSA for 1 h at room temperature. Following several rinses with PBS, cells were incubated with a mix of secondary goat anti-mouse (Alexa Fluor 555 conjugated, dilution 1:1000) and goat anti-rabbit (Alexa Fluor 488 conjugated, dilution 1:600; both from Life Technologies, USA) diluted in PBS with 1% BSA for 1 h at room temperature. Coverslips were then rinsed several times with PBS and mounted on microscope slides with ProLong Gold medium (Life Technologies, USA) with DAPI for DNA counter-staining. Cells were viewed and imaged using Nikon Eclipse Ni-U microscope (Nikon, Japan) equipped with a high definition camera ProgRes MFcool (Jenoptik AG, Germany). Filter sets used were UV-2E/C (340–380 nm excitation and 435–485 nm emission), B-2E/C (465–495 nm excitation and 515–555 nm emission) and Y-2E/C (540–580 nm excitation and 600–660 nm emission). At least 200 cells per data point were imaged. Foci were enumerated using Focicounter (http://focicounter.sourceforge.net/). Integral foci fluorescence was measured using DARFI (https://github.com/varnivey/darfi).

### Statistical analysis

Statistical and mathematical analyses of the data were conducted using the Statistica 8.0 software (StatSoft). Data points in Figures are mean values obtained from three independent experiments; error bars are standard errors. Statistical significance was tested using the Student *t*-test at *p* < 0.05.
